# Perioperative factors associated with intraoperative circulatory decompensation during lung transplantation under initial VV-ECMO support: an exploratory single-center study

**DOI:** 10.1186/s12893-026-04008-6

**Published:** 2026-07-04

**Authors:** Haoshuai Yang, Yang Hao, Shuoyan An, Jin Zhang, Weijie Zhu, Kunsong Su, Chaoyang Liang, Jing Li

**Affiliations:** 1 Department of Thoracic Surgery, China-Japan Friendship Hospital, Beijing, 100029 People’s Republic of China; 2 Department of Cardiology China-Japan Friendship Hospital, Beijing, 100029 People’s Republic of China

**Keywords:** End-stage lung disease, Lung transplantation, Circulatory decompensation

## Abstract

**Background:**

Intraoperative circulatory decompensation during lung transplantation is a life-threatening event that often necessitates a challenging transition from veno-venous (VV) to veno-arterial (VA) extracorporeal membrane oxygenation (ECMO). This study evaluated perioperative variables associated with decompensation under an explicitly exploratory observational framework.

**Methods:**

A retrospective analysis was conducted on 97 patients with end-stage lung disease undergoing lung transplantation with initial VV-ECMO support from January 2020 to December 2022. Intraoperative circulatory decompensation was redefined using objective hemodynamic and echocardiographic criteria; VV-to-VA conversion was recorded as a management consequence rather than as a defining criterion. Propensity score matching (PSM) was applied to balance confounding factors, resulting in 75 analyzed patients. Candidate models were revised as exploratory penalized models and internally assessed by repeated fivefold cross-validation and bootstrap optimism correction.

**Results:**

In the matched descriptive comparison, pH and lactate remained robustly different after FDR correction, while highest intraoperative PASP, pulmonary valve VMAX, aortic valve VMAX, and heart rate were interpreted as exploratory signals. To reduce temporal bias, we separated a preoperative/peri-induction model from a secondary intraoperative monitoring model. In internal validation, the preoperative/peri-induction model achieved a repeated fivefold cross-validation AUC of 0.803 and a bootstrap optimism-corrected AUC of 0.813, with a Brier score of 0.151. The intraoperative monitoring model, which included highest intraoperative PASP, achieved a repeated fivefold cross-validation AUC of 0.853 and a bootstrap optimism-corrected AUC of 0.865, with a Brier score of 0.121.

**Conclusions:**

Lower preoperative arterial pH, higher lactate, and dynamic intraoperative pulmonary pressure changes were associated with clinically documented circulatory decompensation in this exploratory single-center cohort. These internally assessed findings require external validation before clinical implementation.

**Supplementary Information:**

The online version contains supplementary material available at https://doi.org/10.1186/s12893-026-04008-6.

## Introduction

Lung transplantation has emerged as a pivotal therapeutic intervention for end-stage lung diseases, with significant advancements achieved globally in recent years [1]. According to data from the International Society for Heart and Lung Transplantation (ISHLT), over the past decade, approximately 4000 lung transplant procedures are performed annually worldwide, markedly improving patients' quality of life and survival rates [2, 3]. However, the complexity of lung transplantation and postoperative complications remain major clinical challenges. Among these, circulatory decompensation is a critical factor affecting patient outcomes, particularly during the intraoperative phase.

The perioperative use of extracorporeal life support (ECLS) has become increasingly important for patients with compromised cardiac function undergoing lung transplantation. Recently, the ISHLT published a comprehensive three-part consensus statement on the perioperative use of ECLS in lung transplantation, providing evidence-based recommendations for preoperative considerations, intraoperative management, and postoperative care [4–6]. These consensus documents emphasize that patients with severe pulmonary hypertension, right ventricular dysfunction, or hemodynamic instability may particularly benefit from ECLS support during the perioperative period. However, significant variability remains in ECLS configuration strategies across centers, with some institutions preferring primary veno-arterial (VA) the extracorporeal membrane oxygenation (ECMO) for high-risk patients, while others initiate veno-venous (VV) ECMO with selective conversion to VA support [7].

In patients undergoing lung transplantation for end-stage lung disease, the incidence of intraoperative circulatory decompensation is notably high [8]. Common manifestations include reduced left ventricular ejection fraction, impaired cardiac motion, decreased myocardial contractility, reduced systemic circulation pressure, right ventricular dilation, and elevated pulmonary circulation pressure, which may even exceed systemic pressure. These pathophysiological changes not only increase surgical risks but also have profound implications for long-term patient outcomes. When intraoperative circulatory decompensation occurs and cannot be effectively corrected with vasoactive or inotropic agents, the medical team often needs to adjust the ECMO support strategy, typically transitioning from VV-ECMO to VA-ECMOto enhance circulatory support. However, this transition poses significant technical challenges for the medical team and may increase the risk of complications, such as bleeding events at surgical sites, in the brain, or in the abdominal cavity, potentially leading to catastrophic outcomes [9, 10].Furthermore, there is currently no standardized definition or evaluation criteria for intraoperative circulatory decompensation in clinical practice, and the timing of ECMO support initiation varies widely among medical centers, lacking consensus guidelines. This inconsistency may lead to variability in treatment efficacy, thereby impacting patient outcomes. Therefore, in-depth research into the risk factors and management strategies for intraoperative circulatory decompensation is crucial for optimizing clinical practices in lung transplantation.

This study retrospectively analyses clinical data from patients with end-stage lung disease who experienced intraoperative circulatory decompensation during lung transplantation, with the goal of identifying perioperative variables associated with this event and evaluating exploratory risk-stratification models. Given the retrospective single-center design, the limited number of events, and the inclusion of intraoperative variables, the findings should be interpreted as hypothesis-generating associations rather than evidence of causality or as a definitive externally validated predictive tool.

## Methods

### Criteria for intraoperative circulatory decompensation

Intraoperative circulatory decompensation was defined as acute hemodynamic deterioration during lung transplantation documented in the anesthesia or operative record and meeting objective hemodynamic and/or echocardiographic criteria despite standard anesthetic, vasoactive, and pulmonary vasodilator management. Objective criteria included: (1) sustained systemic hypotension, defined as mean arterial pressure < 65 mmHg or systolic arterial pressure < 90 mmHg for at least 5 min; (2) new or worsening right ventricular failure on transesophageal echocardiography, including right ventricular dilation, impaired systolic motion, or interventricular septal shift; (3) pulmonary arterial pressure exceeding systemic arterial pressure or an abrupt clinically significant rise in pulmonary artery systolic pressure during pulmonary artery clamping or reperfusion; (4) refractory need for escalation of vasoactive or inotropic support; and/or (5) evidence of impaired systemic perfusion such as worsening acidosis or rising lactate. VV-to-VA ECMO conversion was recorded as a management consequence of decompensation under our institutional practice and was not itself used as a defining component of the endpoint. The revised objective criteria and escalation pathway are summarized in Supplementary Fig. 1.

### Patientselection

A retrospective analysis was conducted on the clinical data of patients who underwent lung transplantation for end-stage lung disease at China-Japan Friendship Hospital between January 2020 and December 2022. Patients fulfill the following inclusion and exclusion criteria were retrospectively analyzed in the present study.

### Inclusion criteria

① Age ≥ 18 years; ② Bilateral lung transplantation performed by the same surgical team for end-stage lung disease; ③ Initiation of VV-ECMO support at the start of surgery.

### Exclusion criteria

① Unilateral lung transplantation; ② Aborted surgery due to intraoperative complications; ③ Incomplete or missing clinical data.

**Fig. 1:**
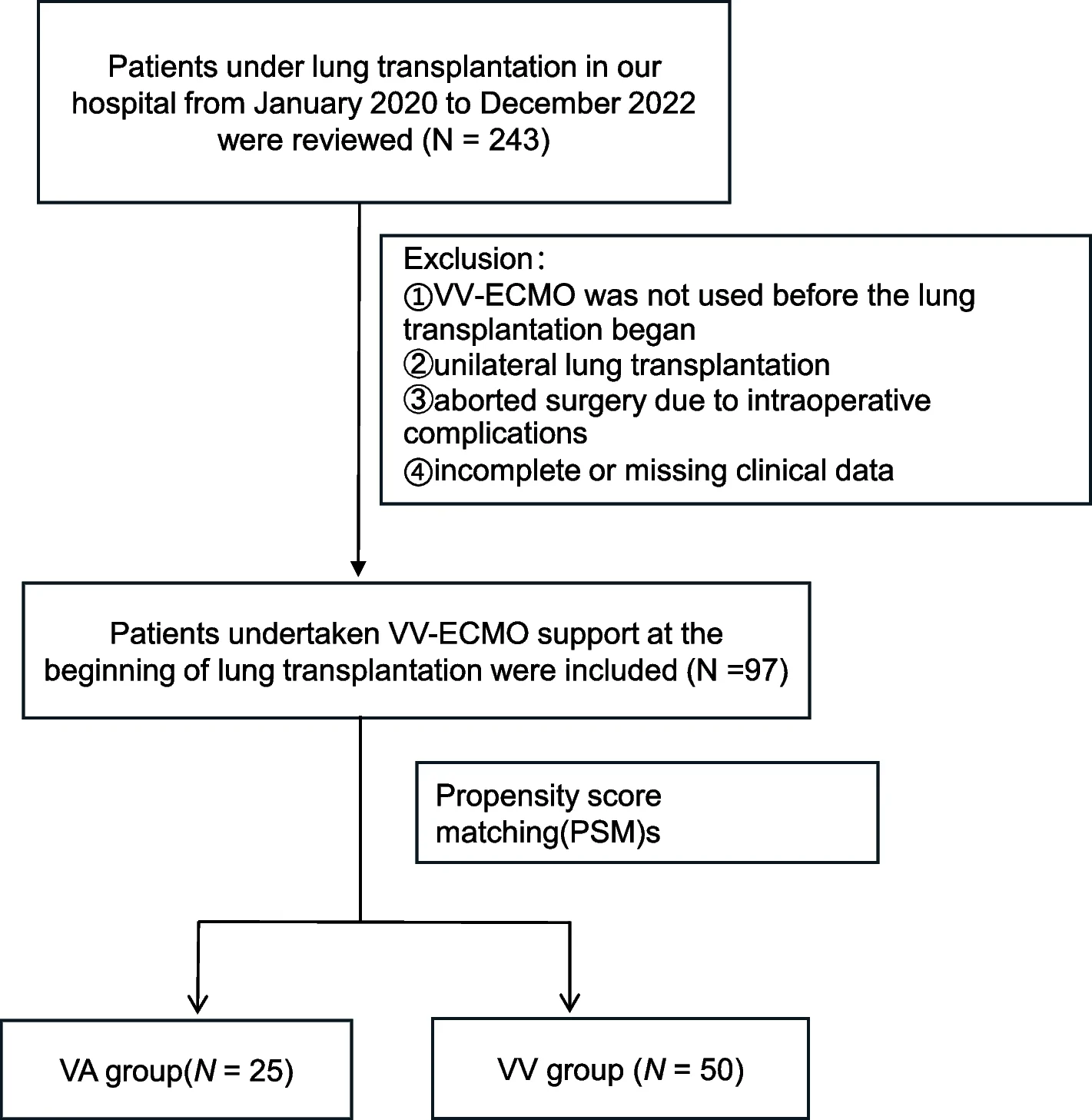
Study Workflow and Patient Selection

The analysis focused on the following parameters for these 97 patients: gender, age, body mass index (BMI), operative time, blood loss, preoperative random arterial blood gas pH, lactate levels, oxygenation index, partial pressure of carbon dioxide (PaCO₂), systolic blood pressure upon entering the operating room, heart rate upon entering the operating room, preoperative cardiac enzyme levels, preoperative echocardiographic parameters, intraoperative changes in pulmonary artery systolic pressure, and parameters from anesthesia monitoring. The workflow of this study is shown in Fig. 1.

### Laboratory parameters

All preoperative laboratory parameters were measured by the Department of Laboratory Medicine at China-Japan Friendship Hospital one day before the surgery. Preoperative echocardiographic parameters were measured by the same echocardiography team within one week before the surgery. Preoperative random arterial blood gas analysis was performed in the lung transplantation ward of China-Japan Friendship Hospital one day before the surgery using a Radiometer ABL9 analyzer.

### Surgical procedure

All lung transplantations were performed by the same surgical team using a standardized protocol. Bilateral sequential lung transplantation was performed through a bilateral posterolateral thoracotomy. The decision regarding which lung to transplant first was based on a comprehensive evaluation of preoperative imaging and clinical assessment. The general principles were: (1) Transplant the more severely diseased lung first—assessed by preoperative CT imaging showing extent of fibrosis, bronchiectasis, or destruction; (2) Transplant the lung with poorer perfusion first—evaluated by perfusion scintigraphy or CT pulmonary angiography showing differential blood flow; (3) Consider technical factors such as presence of pleural adhesions, previous thoracic surgery, or anatomical variations.

### Intraoperative fluid and pulmonary pressure management

Intraoperative fluid and pulmonary pressure management was guided by pulmonary artery catheter measurements, with a goal of maintaining euvolemia [11]. Fluid administration was conservative, with crystalloid infusion rates typically maintained at 2–4 mL/kg/hour, and colloid solutions used selectively for volume expansion when indicated.

Pulmonary pressure was managed with inhaled nitric oxide initiated at 10–20 ppm during the procedure. Systemic hypotension was treated with norepinephrine and/or vasopressin to maintain mean arterial pressure. In cases of right ventricular dysfunction, milrinone was used for inotropic support. High-dose vasoactive drugs were avoided when possible, as they can increase pulmonary vascular resistance and exacerbate right heart strain [12]. The highest intraoperative pulmonary artery systolic pressure value recorded by the catheter was used for analysis. Heart rate and systolic blood pressure upon entering the operating room were measured by the anesthesia machine at the beginning of anesthesia.

### Patient grouping and propensity score matching (PSM)

To control for major baseline differences in descriptive comparisons, propensity score matching analysis was used. Propensity scores were estimated using sex, age, primary disease type, BMI, pulmonary artery systolic pressure at the beginning of surgery, and systolic blood pressure at the beginning of surgery. Patients in the VA-conversion and VV-maintained groups were matched at a 1:2 ratio using nearest-neighbor matching without replacement. No caliper was applied in the original matched analysis. Balance was assessed using standardized mean differences (SMDs), with absolute SMD values < 0.10 considered optimal and values > 0.20 considered potentially meaningful residual imbalance. The post-matching SMD table is reported in Table 1 and Supplementary Fig. 2; residual imbalance in pulmonary artery systolic pressure at the beginning of surgery (SMD approximately 0.246) was acknowledged in the interpretation.

**Table 1:** Basic information and clinical characteristics of patients

Variable	Group	Before PSM (*N* = 97)	Statistic	*P*-value	SMD	After PSM (*N* = 75)	Statistic	*P*-value	SMD
Age (Mean ± SD)	Total	54.08(12.24)				55.91 (11.37)			
	VV group	55.60(10.27)	t = 0.914	0.365	0.191	55.60 (10.27)	t = −0.187	0.852	−0.044
	VA group	53.28(12.71)				56.13 (12.14)			
Systemic Systolic Pressure (Mean ± SD)	Total	133.71(23.50)				131.84 (22.82)			
	VV group	129.24(17.50)	t = −1.333	0.188	−0.261	129.24(17.50)	t = −0.774	0.442	−0.180
	VA group	135.35(25.08)				133.46(26.14)			
Sex-Male (N, %)	Total	66 (68.04%)				52 (69.33%)			
	VV group	18 (72.00%)	x^2^ = 0.059	0.807	0.113	18 (72.00%)	x^2^ = 0.000	1.000	0.059
	VA group	48 (66.67%)				34 (68.00%)			
The pulmonary artery systolic pressure at the beginning of surgery (Mean ± SD)	Total	38.07(10.59)				38.64 (11.30)			
	VV group	40.11 (12.51)	t = 1.004	0.322	0.265	40.11 (12.51)	t = 0.844	0.403	0.246
	VA group	37.35 (9.56)				37.55 (10.71)			
BMI (Mean ± SD)	Total	22.24 (4.25)				22.16 (4.39)			
	VV group	22.11 (4.96)	t = −0.150	0.882	−0.039	22.11 (4.96)	t = −0.070	0.945	−0.020
	VA group	22.28 (3.98)				22.19 (4.06)			

### Statistical analysis

Continuous variables were tested for normality and homoscedasticity; normally distributed data are expressed as mean ± standard deviation, while non-normally distributed data are presented as median [interquartile range (IQR)]. Categorical variables are reported as frequencies and percentages. Differences between groups were analyzed using appropriate parametric or non-parametric tests. Benjamini–Hochberg false discovery rate (FDR) correction was applied and q-values were reported alongside unadjusted p-values. Results from univariate testing were interpreted as descriptive and hypothesis-generating rather than as causal evidence.

Given the narrow range of pH values, we scaled pH by dividing by 0.1 to obtain clinically interpretable odds ratios. Because the cohort included only 25 events, ordinary multivariable logistic regression with multiple predictors was considered vulnerable to overfitting and sparse-data instability. We therefore revised the modeling framework to emphasize penalized regression and internal validation. A primary preoperative/peri-induction model included variables available before or at the beginning of surgery (pH, lactate, heart rate at the beginning of surgery, pulmonary valve VMAX, and PASP at the beginning of surgery). A secondary intraoperative monitoring model included highest intraoperative PASP and was not interpreted as a preoperative prediction model because this variable occurs during the at-risk period. Model performance was assessed by repeated fivefold cross-validation and bootstrap optimism correction. Calibration was assessed using Brier score and calibration plots. Sensitivity and specificity at the selected cutoff were estimated from internally validated predictions rather than from the apparent training-set fit.

## Results

### Patient characteristics

During the study period from January 2020 to December 2022, a total of 243 patients underwent lung transplantation at our institution. Among these, 109 patients (44.9%) were planned for VV-ECMO support at the beginning of surgery based on our institutional protocol. After applying inclusion and exclusion criteria, 97 patients were ultimately included in the analysis.Among these, 25 required intraoperative conversion to VA-ECMO due to circulatory decompensation (VA group). The remaining 72 patients maintained VV-ECMO throughout surgery (VV group). After propensity score matching, 75 lung transplant recipients were included in this study: 50 in the VV group and 25 in the VA group. Baseline characteristics of both groups are summarized in Table 1. Matching improved balance for most prespecified covariates, although residual imbalance remained for PASP at the beginning of surgery. (Supplementary Fig. 2).

### Differences in variable characteristics between VV group and VA group

Table 2 presents the baseline characteristics of the study population after propensity score matching. The primary disease distribution was similar between the VV group (*N* = 50) and VA group (*N* = 25), with no statistically significant difference (*p* = 0.79). Interstitial lung disease was the predominant diagnosis in both groups (72% in each group). Other diagnoses included COPD (6% vs. 12%), bronchiolitis obliterans (8% vs. 4%), bronchiectasis (8% vs. 8%), pneumoconiosis (2% vs. 4%), and others (4% vs. 0%). The comparable disease distribution between groups suggests that the requirement for VA conversion was not primarily driven by differences in underlying disease etiology.

**Table 2:** Comparison of Variable Differences between VV Group and VA Group

**Variable**		**VV (*N* = 50)**	**VA (*N* = 25)**	***P*-value**	**FDR q-value**
Primary disease	N(%)			0.790	0.885
Interstitial lung disease		36 (72%)	18 (72%)		
COPD		3 (6%)	3 (12%)		
Bronchiolitis obliterans		4 (8%)	1 (4%)		
Bronchiectasis		4 (8%)	2 (8%)		
Pneumoconiosis		1 (2%)	1 (4%)		
Others		2 (4%)	0 (0%)		
Operative time (min)	Mean ± SD	405.00 (78.68)	491.88 (81.57)	< 0.001	0.005
Bleeding volume (ml)	Median (IQR)	500.00 [400.00, 800.00]	1000.00 [600.00, 1800.00]	< 0.001	0.005
TNI (ng/ml)	Median (IQR)	0.01 [0.00, 0.02]	0.01 [0.00, 0.02]	0.256	0.551
CK-MB (ng/ml)	Median (IQR)	1.50 [0.80, 2.18]	1.00 [0.70, 1.80]	0.210	0.551
Myo (ng/ml)	Median (IQR)	16.70 [10.20, 23.05]	18.00 [12.00, 25.60]	0.504	0.827
BNP (pg/ml)	Median (IQR)	39.90 [23.00, 67.75]	28.00 [17.00, 133.00]	0.629	0.869
The ascending aorta diameter (mm)	Median (IQR)	32.15 [29.25, 35.00]	32.00 [30.00, 33.00]	0.457	0.827
The interventricular septum thickness (mm)	Median (IQR)	9.00 [8.00, 10.00]	9.01 [8.00, 10.00]	0.714	0.869
The left ventricular end-systolic diameter (mm)	Mean ± SD	42.78 (4.88)	43.29 (5.83)	0.689	0.869
Ejection fraction (%)	Mean ± SD	27.45 (3.74)	27.46 (4.13)	0.986	0.986
The left ventricular longitudinal fractional shortening (%)	Mean ± SD	35.89 (4.59)	37.24 (4.63)	0.237	0.551
The right atrial transverse diameter (mm)	Median (IQR)	34.50 [30.00, 40.75]	37.00 [34.00, 41.00]	0.145	0.451
The right ventricular basal segmental transverse diameter (mm)	Median (IQR)	33.00 [30.00, 38.00]	36.00 [32.00, 39.00]	0.226	0.551
Lactic acid (mmol/L)	Median (IQR)	1.20 [0.80, 1.60]	2.00 [1.30, 3.60]	0.001	0.007
PH	Median (IQR)	7.41 [7.37, 7.43]	7.33 [7.27, 7.38]	< 0.001	0.005
The pulmonary artery systolic pressure at the beginning of surgery (mmHg)	Mean ± SD	38.47 (10.65)	40.11 (12.51)	0.557	0.827
The highest pulmonary artery systolic pressure (mmHg)	Mean ± SD	65.43 (9.92)	73.05 (18.12)	0.021	0.084
Blood transfusion volume (ml)	Median (IQR)	400.00 [0.00, 800.00]	400.00 [0.00, 800.00]	0.972	0.986
Infusion volume (ml)	Median (IQR)	3350.00 [2400.00, 4350.00]	3400.00 [2600.00, 4200.00]	0.857	0.923
Urine volume (ml)	Median (IQR)	725.00 [500.00, 1275.00]	600.00 [500.00, 1000.00]	0.307	0.614
Hemoglobin (g/dL)	Mean ± SD	12.26 (1.63)	12.52 (1.64)	0.528	0.827
The maximum dose of norepinephrine (ug/kg/min)	Median (IQR)	0.03 [0.02, 0.05]	0.02 [0.02, 0.08]	0.66	0.869
The oxygenation index at the beginning of surgery (mmHg)	Mean ± SD	219.42 (90.58)	231.16 (60.91)	0.561	0.827
PCO2 at the beginning of surgery (mmHg)	Median (IQR)	45.00 [41.25, 51.50]	44.00 [43.00, 49.00]	0.770	0.885
The heart rate at the beginning of surgery (BMP)	Mean ± SD	85.34 (15.69)	94.76 (18.72)	0.025	0.088
The maximum blood flow velocity above aortic valve (cm/s)	Median (IQR)	110.50 [102.00, 122.75]	121.00 [110.00, 133.00]	0.021	0.084
The maximum blood flow velocity above pulmonary valve (cm/s)	Median (IQR)	79.50 [71.25, 87.75]	86.00 [80.00, 99.00]	0.010	0.056

*Abbreviations*: *FDR* false discovery rate, *IQR* interquartile range, *PASP* pulmonary artery systolic pressure, *VMAX* maximum blood flow velocity. FDR *q*-values were calculated using the Benjamini–Hochberg procedure across the univariate comparisons in Table 2

Intraoperatively, the VA group demonstrated significantly longer operative duration (491.88 ± 81.57 vs. 405.00 ± 78.68 min, p < 0.001) and higher intraoperative blood loss (median: 1000 mL [IQR 600–1800] vs. 500 mL [IQR 400–800], p < 0.001), but were interpreted as intraoperative consequences or correlates rather than baseline predictors. After Benjamini–Hochberg FDR correction for multiple comparisons, pH and lactate remained robustly different between groups, acid–base metabolic analysis revealed elevated lactate levels (2.00 mmol/L [1.30–3.60] vs. 1.20 mmol/L [0.80–1.60], *p* = 0.001) and reduced pH values (7.33 [7.27–7.38] vs. 7.41 [7.37–7.43], p < 0.001) in the VA group, indicating more severe metabolic acidosis. Exploratory echocardiographic and peri-induction variables, including highest intraoperative PASP, pulmonary valve VMAX, aortic valve VMAX, and heart rate at the beginning of surgery, were higher in the VA group but did not remain significant after FDR correction. No significant differences were observed in echocardiographic parameters (right atrial/ventricular dimensions, ejection fraction, left ventricular end-systolic diameter) or myocardial injury biomarkers (TNI, CK-MB, Myo, BNP), transfusion volume, fluid intake, or hemoglobin levels. The residual post-matching imbalance in PASP at the beginning of surgery was also considered when interpreting the matched comparison.

### Detailed characteristics of VV-to-VA conversion events

**Fig. 2:**
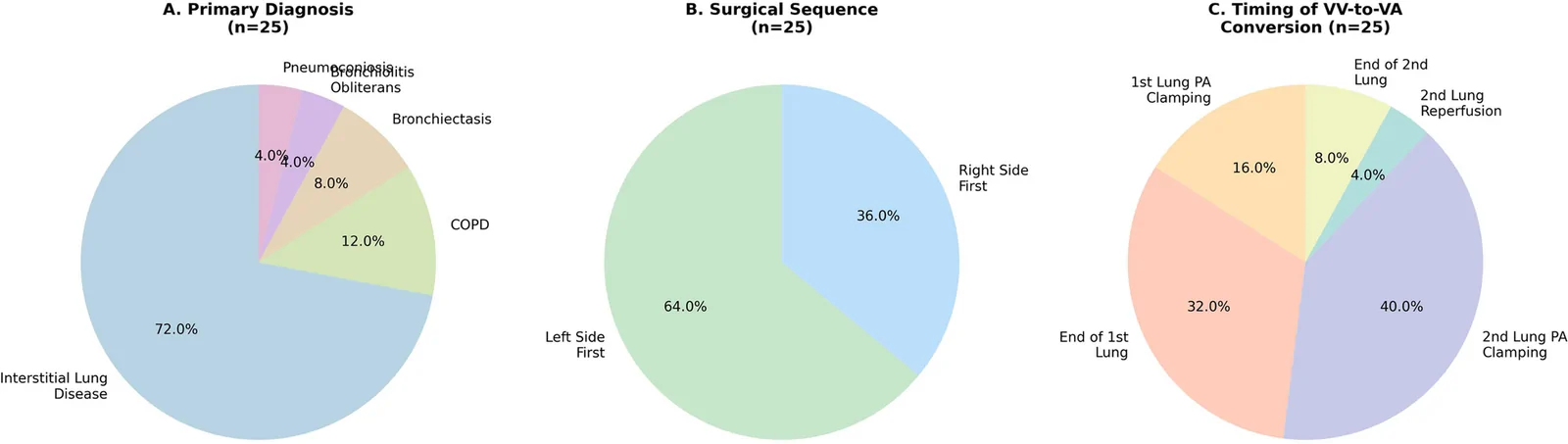
Clinical Characteristics of Study Population. Pie charts depicting the distribution of clinical variables among patients requiring VV-to-VA conversion. **A** Primary diagnosis categories; **B** Surgical sequence (left versus right side first); and **C** Timing of conversion events during the surgical procedure

To provide granular insight into the clinical scenarios leading to intraoperative circulatory decompensation, we conducted a detailed analysis of all 25 VA group patients (Supplementary Table 1, Fig. 2).

Among the 25 patients requiring VA conversion, the left lung was transplanted first in 16 patients (64.0%), while the right lung was transplanted first in 9 patients (36.0%). Conversion events were distributed throughout the surgical procedure as follows: during second lung pulmonary artery clamping in 10 patients (40.0%); at the end of the first lung procedure in 8 patients (32.0%); during first lung pulmonary artery clamping in 4 patients (16.0%); at the end of the second lung procedure in 2 patients (8.0%); and during second lung reperfusion in 1 patient (4.0%). Notably, 72% of conversions (*N* = 18) occurred during or immediately after the first lung procedure, highlighting the critical hemodynamic stress during initial graft implantation and the importance of vigilant monitoring during this phase.

The primary triggers for conversion, documented in operative records, included: pulmonary artery systolic pressure elevation with concurrent systemic circulatory failure in 22 patients (88.0%), with documented PASP ranging from 50 to 108 mmHg (mean 76.5 ± 15.2 mmHg); acute right ventricular failure evidenced by TEE in 2 patients (8.0%); primary graft dysfunction with pulmonary edema in 3 patients (12.0%); massive bleeding with refractory hypotension in 1 patient (4.0%); and refractory hypotension despite maximal vasoactive support in 4 patients (16.0%). Multiple concurrent triggers were present in most patients, reflecting the complex, multifactorial nature of intraoperative circulatory decompensation in this high-risk population.

### Perioperative variables associated with circulatory decompensation

Univariate logistic regression analyses identified perioperative variables associated with circulatory decompensation (Table 3). Univariate analysis revealed associations with lactate levels (OR = 1.74, 95% CI: 1.09–2.75, *p* = 0.019), pH (OR = 0.19, 95% CI: 0.079–0.432, p < 0.001), The highest value of pulmonary artery systolic pressure (OR = 1.04, 95% CI: 1.00–1.09, *p* = 0.031), pulmonary valve VMAX (OR = 1.04, 95% CI: 1.01–1.07, *p* = 0.010), and The heart rate at the beginning of surgery (OR = 1.03, 95% CI: 1.00–1.06, *p* = 0.047).

**Table 3:** Results of univariate analysis of various variables

Varible	OR (univariable)	*P*-Value
TNI (ng/ml)	0.00 (0.00–732444.52)	*p* =.450
CK-MB (ng/ml)	0.84 (0.59–1.18)	*p* =.315
Myo (ng/ml)	1.00 (0.98–1.03)	*p* =.736
BNP (pg/ml)	1.00 (1.00–1.00)	*p* =.183
The ascending aorta diameter (mm)	0.94 (0.83–1.07)	*p* =.358
The interventricular septum thickness (mm)	1.03 (0.74–1.43)	*p* =.865
The left ventricular end-systolic diameter (mm)	1.00 (0.88–1.14)	*p* =.986
Ejection fraction (%)	1.03 (0.95–1.12)	*p* =.419
The left ventricular longitudinal fractional shortening (%)	0.98 (0.88–1.10)	*p* =.746
The right atrial transverse diameter (mm)	1.04 (0.98–1.12)	*p* =.208
The right ventricular basal segmental transverse diameter (mm)	1.03 (0.97–1.10)	*p* =.365
Lactic acid (mmol/L)	1.74 (1.09–2.75)	*p* =.019
PH	0.19 (0.08–0.43)	P <.001
The pulmonary artery systolic pressure at the beginning of surgery (mmHg)	1.01 (0.97–1.06)	*p* =.551
The highest value of pulmonary artery systolic pressure (mmHg)	1.04 (1.00–1.09)	*p* =.031
Blood transfusion volume (ml)	1.00 (1.00–1.00)	*p* =.824
Infusion volume (ml)	1.00 (1.00–1.00)	*p* =.353
Urine volume (ml)	1.00 (1.00–1.00)	*p* =.215
Hemoglobin (g/dL)	1.10 (0.82–1.48)	*p* =.523
The maximum blood flow velocity above aortic valve (cm/s)	1.02 (1.00–1.04)	*p* =.083
The maximum blood flow velocity above pulmonary valve (cm/s)	1.03 (1.00–1.07)	*p* =.029
The oxygenation index at the beginning of surgery (mmHg)	1.00 (1.00–1.01)	*p* =.555
The partial pressure of carbon dioxide at the beginning of surgery (mmHg)	0.99 (0.95–1.03)	*p* =.474
The heart rate at the beginning of surgery (BMP)	1.03 (1.00–1.06)	*p* =.047

### Predictive performance of perioperative variables

ROC curve analysis evaluated the predictive efficacy of significant variables (Supplementary Fig. 3). Metabolic markers demonstrated high descriptive ROC performance: pH (AUC = 0.796, sensitivity 92.0%, specificity 64.0%) and lactate (AUC = 0.734, sensitivity 78.0%, specificity 60.0%) were robust early warning indicators. Hemodynamic parameters showed moderate performance: peak pulmonary artery systolic pressure (AUC = 0.663, sensitivity 80.0%, specificity 60.0%) and pulmonary valve VMAX (AUC = 0.682, sensitivity 60.0%, specificity 72.0%). The heart rate at the beginning of surgery exhibited limited utility (AUC = 0.641, sensitivity 90.0%, specificity 32.0%). These findings suggest that pH and lactate should be considered for perioperative monitoring, while pulmonary artery systolic pressure parameters provide supplementary guidance for clinical using.

### Ridge-penalized logistic regression model construction

Ridge-penalized logistic regression was used to shrink coefficients and reduce instability while retaining clinically prespecified variables in each model. A primary preoperative/peri-induction model included pH, lactate, heart rate at the beginning of surgery, pulmonary valve VMAX, and PASP at the beginning of surgery. A secondary intraoperative monitoring model included pH, lactate, heart rate at the beginning of surgery, pulmonary valve VMAX, and highest intraoperative PASP. Internal validation metrics are presented in Supplementary Table 2, and penalized regression coefficients are presented in Supplementary Table 3. Highest intraoperative PASP was included only in the secondary model because it is measured during the at-risk intraoperative period.

All predictors were standardized before model fitting. The means and standard deviations used for standardization were: pH (mean 7.376, SD 0.078); lactate (mean 2.204, SD 2.970); heart rate mean (90.072, SD 17.484); pulmonary valve VMAX (mean 81.283, SD 21.659); PASP at the beginning of surgery (mean 38.060, SD 10.403); and highest intraoperative PASP (mean 66.153, SD 13.219).

### Internal validation and model performance

**Fig. 3:**
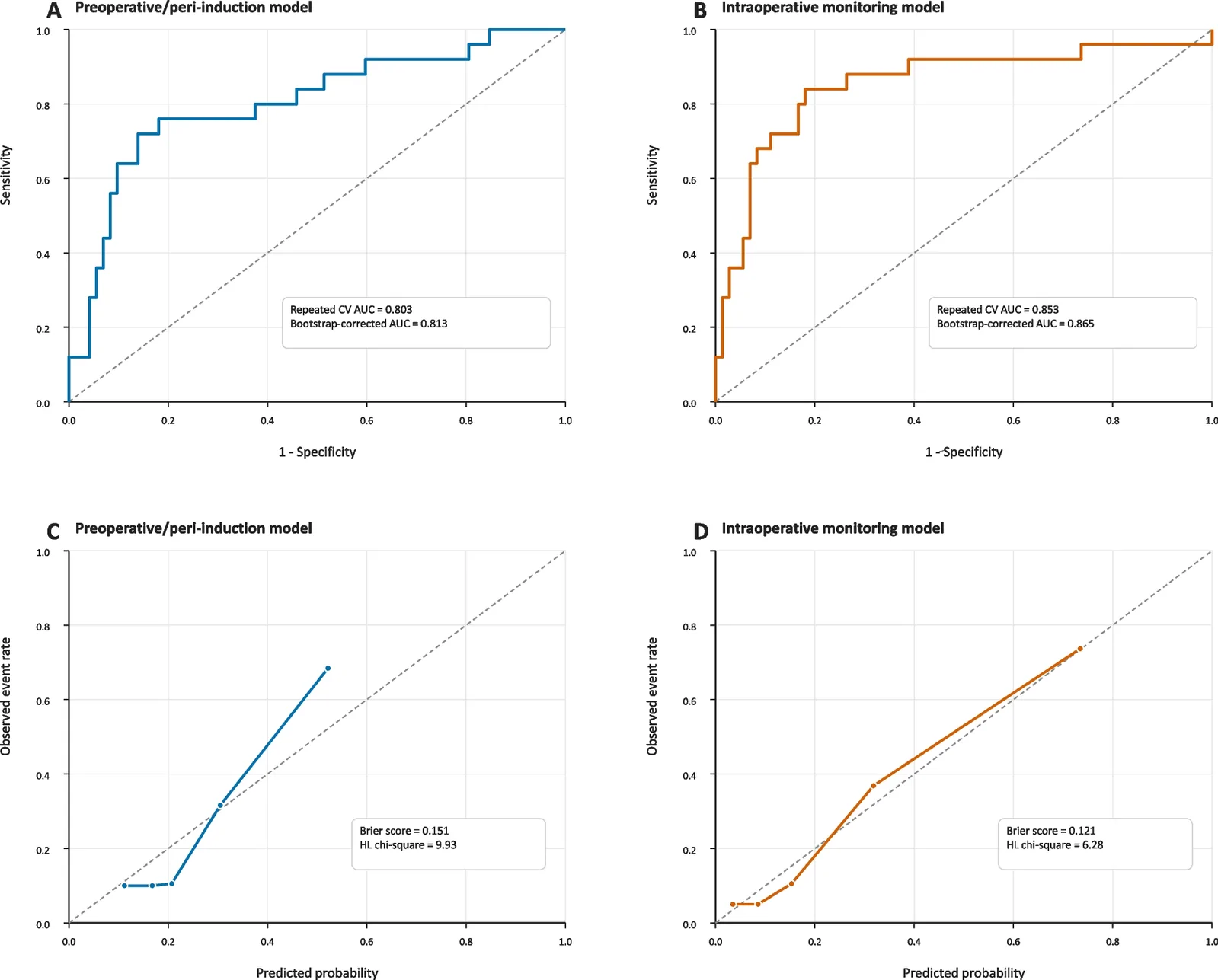
Internally Validated Ridge-Penalized Model Performance. **A** Receiver operating characteristic curve for the primary preoperative/peri-induction ridge-penalized model. **B** Receiver operating characteristic curve for the secondary intraoperative monitoring model. **C** Calibration plot for the primary preoperative/peri-induction model. **D** Calibration plot for the secondary intraoperative monitoring model. All curves were based on internally validated predictions from repeated fivefold cross-validation

To avoid optimistic interpretation of apparent training-set performance, model performance was evaluated using repeated fivefold cross-validation and bootstrap optimism correction. The primary preoperative/peri-induction model achieved a repeated fivefold cross-validation AUC of 0.803 and a bootstrap optimism-corrected AUC of 0.813 (Fig. 3A). The corresponding Brier score was 0.151, and the calibration plot is shown in Fig. 3C. Using internally validated predictions, the selected cutoff was 0.318, with sensitivity of 72.0% and specificity of 86.1%.

The secondary intraoperative monitoring model achieved a repeated fivefold cross-validation AUC of 0.853 and a bootstrap optimism-corrected AUC of 0.865 (Fig. 3B), with a Brier score of 0.121 and calibration shown in Fig. 3D. At the internally validated cutoff of 0.269, sensitivity was 80.0% and specificity was 84.7%. Because highest PASP is measured during the at-risk period, this model should be interpreted only as an intraoperative monitoring framework and not as a preoperative prediction model. The distributions of internally validated predicted probabilities for the two models are provided in Supplementary Fig. 4.

## Discussion

This exploratory single-center study found that lower preoperative arterial pH and higher lactate were consistently associated with clinically documented intraoperative circulatory decompensation under initial VV-ECMO support. Dynamic pulmonary pressure variables, particularly highest intraoperative PASP, carried additional information during surgery, but they should be interpreted as intraoperative monitoring markers rather than preoperative predictors. These results support cautious perioperative risk stratification and hypothesis generation, not causal inference or immediate clinical implementation.

Our findings can be meaningfully contextualized within the framework of the recently published ISHLT consensus statements on perioperative ECLS use in lung transplantation [4–6]. The preoperative considerations part identifies severe pulmonary hypertension, elevated right atrial pressure, and RV dysfunction as key risk factors warranting preoperative ECLS planning—consistent with our observation that highest intraoperative PASP functioned as a secondary intraoperative monitoring marker associated with decompensation [13]. Our revised analysis is consistent with this general framework but should not be interpreted as providing a validated decision rule. Instead, it suggests that metabolic vulnerability and dynamic right ventricular afterload may identify patients who warrant heightened intraoperative monitoring.

The choice of initial ECLS configuration remains a subject of debate in the lung transplantation community. Our center's preference for initiating VV-ECMO with selective conversion to VA-ECMO is based on several considerations supported by the literature. VV-ECMO ensures adequate gas exchange while preserving native cardiac output and pulsatile arterial flow, maintaining coronary perfusion with oxygenated blood and enabling assessment of intrinsic hemodynamic reserve during early surgical phases [14]. Bernhardt et al. [15] noted in the ISHLT/HFSA Guidelines that bleeding complications in mechanical circulatory support are common and frequently necessitate withdrawal of anticoagulation. Kirklin et al. [16] similarly recommended low or no heparin regimes for patients with significant adhesions and impaired coagulation status. However, any discussion of potential advantages of VV-ECMO, such as avoidance of arterial cannulation, preservation of pulsatile flow, or possibly lower vascular-complication risk, should be interpreted as background rationale rather than evidence generated by the present study. Comparative effectiveness studies are needed to determine which patients may benefit from primary VA-ECMO rather than VV-first support.

In previous studies, mean pulmonary artery pressure (mPAP) has been consistently identified as a prognostic marker for long-term outcomes in lung transplant recipients [17, 18], particularly in cohorts dominated by idiopathic pulmonary fibrosis where chronic pulmonary vascular remodeling is the predominant pathophysiological feature [19, 20]. These studies primarily focused on mortality and long-term survival, outcomes that are largely influenced by sustained elevations in pulmonary vascular resistance, of which mPAP is a representative measure. In this context, highest intraoperative PASP was associated with decompensation in the secondary monitoring model; however, because it is measured during the at-risk period, it may partly reflect the evolution of the event itself. Similarly, pulmonary valve VMAX may reflect right ventricular afterload or pulmonary vascular properties, but the present retrospective dataset cannot establish a mechanistic pathway. These variables are therefore best viewed as dynamic monitoring markers that may help identify impending instability during surgery.

In addition to the prognostic value of systolic pulmonary artery pressure, our study also identified baseline heart rate as an associated variable of intraoperative circulatory decompensation. Elevated resting heart rate in patients with end-stage lung disease often reflects a state of increased sympathetic activation and impaired cardiac autonomic regulation [21]. The association between baseline heart rate and intraoperative circulatory decompensation should be interpreted with careful clinical insight, an elevated resting heart rate may not necessarily act as a direct causal risk factor, but rather as a risk marker that reflects an underlying adverse physiological condition such as heightened sympathetic activation, systemic inflammation, hypoxemia, anemia, or subclinical infection. In this retrospective dataset, its association did not remain robust after multiple-comparison correction and should therefore be interpreted cautiously.

Our findings highlight preoperative arterial pH and lactate as candidate markers of latent metabolic and microcirculatory dysfunction. Lactate, traditionally considered a marker of hypoxia and tissue malperfusion [22], can also rise through aerobic glycolysis in pulmonary vascular remodeling and failing myocardium, particularly in pulmonary hypertension and interstitial lung disease [23]. Acidosis impairs myocardial contractility, vascular tone, renal function, and microcirculatory perfusion, thereby diminishing the cardiopulmonary adaptive reserve [24, 25]. Importantly, even mild lactate elevationsare associated with microcirculatory failure and clinical deterioration [26]. In our study, patients who ultimately required intraoperative VA-ECMO exhibited significantly lower pH and higher lactate levels preoperatively, despite comparable echocardiographic and biomarker profiles, suggesting that metabolic markers may detect occult circulatory vulnerability more sensitively than conventional cardiac assessments. This aligns with previous findings in cardiac, liver, and lung transplantation, where elevated lactate has been associated with ECMO need, graft dysfunction, acute kidney injury, and prolonged mechanical ventilation [27–29].

From a methodological perspective, ridge-penalized regression and internal validation reduced, but did not eliminate, the risk of overfitting. The internally validated performance estimates were lower than the apparent training-set values, underscoring the importance of avoiding overinterpretation. The models should be considered preliminary risk-stratification frameworks pending external validation in a larger prospective cohort.

This study has several important limitations that should be acknowledged. First, as a single-center retrospective study with a relatively small sample size, the analysis is vulnerable to selection bias, limited statistical power, overfitting, and sparse-data instability. Therefore, our findings should be considered as exploratory hypotheses rather than definitive risk factors, and the candidate markers require validation in larger, multicenter prospective cohorts before implementation in clinical practice. Second, although we revised the endpoint definition to emphasize objective hemodynamic and echocardiographic criteria, retrospective documentation may be incomplete, particularly among patients who did not undergo conversion. Third, highest intraoperative PASP is temporally downstream of surgical start and may occur during the evolution of decompensation; therefore, analyses including this variable are subject to temporal leakage and should be interpreted only within an intraoperative monitoring framework. Fourth, PSM reduced but did not eliminate baseline imbalance; PASP at the beginning of surgery remained imbalanced after matching. Fifth, no external validation cohort was available, and all discrimination, calibration, and cutoff estimates are internally validated only. Additionally, the decision to convert from VV to VA involves subjective clinical judgment that may vary among practitioners, introducing potential bias in outcome classification. Sixth, we did not include postoperative outcomes in this analysis, which would strengthen the clinical relevance of our findings.

Despite these limitations, the study provides preliminary data on perioperative variables associated with intraoperative circulatory decompensation. Future studies should prospectively validate objective decompensation criteria, test separate preoperative and intraoperative monitoring models, incorporate advanced right-heart functional measures, evaluate postoperative outcomes, and compare ECLS configuration strategies across centers.

## Conclusions

In conclusion, lower preoperative arterial pH, higher lactate, and intraoperative pulmonary pressure escalation were associated with circulatory decompensation during lung transplantation under initial VV-ECMO support. Highest intraoperative PASP should be interpreted as a dynamic monitoring marker rather than a baseline predictor. The findings are exploratory and require prospective external validation before they can guide standardized ECMO strategy selection.

## Supplementary Information

Supplementary Material 1: Supplementary Figure 1. Work Flow of Patient Selection. Supplementary Figure 2. Balance Assessment Before and After Propensity Score Matching. Supplementary Figure 3. Predictive Performance of Perioperative Variables. Supplementary Figure 4. Distribution of Internally Validated Predicted Probabilities 

## Data Availability

The original data presented in the study are included in the article, which are available from the corresponding author upon reasonable request.

## Abbreviations

AUC: Area under the ROC curve
BMI: Body mass index
BNP: B-type natriuretic peptide
CK-MB: Creatine kinase-MB
ECMO: Extracorporeal membrane oxygenation
ECLS: Extracorporeal Life Support
IQR: Interquartile range
ISHLT: International Society for Heart and Lung Transplantation
LVEF: Left ventricular ejection fraction
PaCO₂: Partial pressure of carbon dioxide
PASP: Pulmonary Artery Systolic Pressure
PSM: Propensity score matching
ROC: Receiver operating characteristic
TNI: Troponin I
VA-ECMO: Veno-arterial extracorporeal membrane oxygenation
VMAX: Maximum blood flow velocity
VV-ECMO: Veno-venous extracorporeal membrane oxygenation
