# Supplementary material for: Perioperative factors associated with intraoperative circulatory decompensation during lung transplantation under initial VV-ECMO support: an exploratory single-center study
Source: BMC Surg. 2026 Jul 4;26:649. doi: 10.1186/s12893-026-04008-6 (PMC13617856; doi:10.1186/s12893-026-04008-6)
Supplement: Supplementary file 1 [file 12893_2026_4008_MOESM1_ESM.zip › supplementary figure 4.pdf]

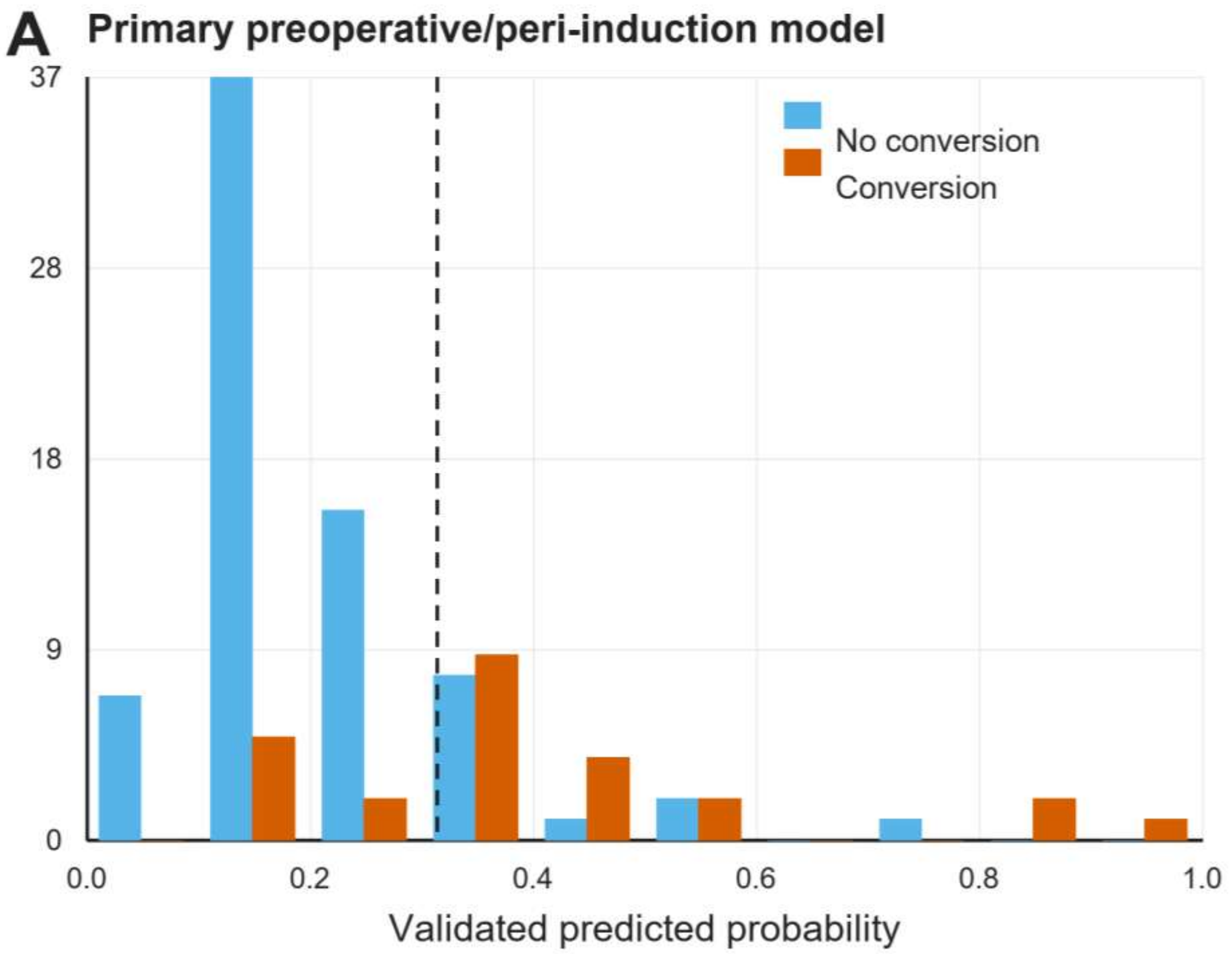
A
Primary preoperative/peri-induction model
37
28
18
9
0
0.0
0.2
0.4
0.6
0.8
1.0
No conversion
Conversion
Validated predicted probability
B
Secondary intraoperative monitoring model
34
26
17
8
0
0.0
0.2
0.4
0.6
0.8
1.0
No conversion
Conversion
Validated predicted probability
