# Supplementary material for: Perioperative factors associated with intraoperative circulatory decompensation during lung transplantation under initial VV-ECMO support: an exploratory single-center study
Source: BMC Surg. 2026 Jul 4;26:649. doi: 10.1186/s12893-026-04008-6 (PMC13617856; doi:10.1186/s12893-026-04008-6)
Supplement: Supplementary file 1 [file 12893_2026_4008_MOESM1_ESM.zip › Supplementary Table 2.docx]

**Supplementary Table 2 Internal validation results of ridge-penalized logistic regression models**

| Model | Candidate variables | Penalty | Repeated 5-fold CV AUC | Bootstrap-corrected AUC | Brier score | Cutoff | Sensitivity | Specificity |
| --- | --- | --- | --- | --- | --- | --- | --- | --- |
| Primary preoperative/peri-induction model | pH; lactate; heart rate at beginning of surgery; pulmonary valve VMAX; PASP at beginning of surgery | 10 | 0.803 | 0.813 | 0.151 | 0.318 | 72.0% | 86.1% |
| Secondary intraoperative monitoring model | pH; lactate; heart rate at beginning of surgery; pulmonary valve VMAX; highest intraoperative PASP | 1 | 0.853 | 0.865 | 0.121 | 0.269 | 80.0% | 84.7% |

*The primary model included only preoperative/peri-induction variables. The secondary model included highest intraoperative PASP and should be interpreted as an intraoperative monitoring model rather than a preoperative prediction model.*
