# Supplementary material for: Perioperative factors associated with intraoperative circulatory decompensation during lung transplantation under initial VV-ECMO support: an exploratory single-center study
Source: BMC Surg. 2026 Jul 4;26:649. doi: 10.1186/s12893-026-04008-6 (PMC13617856; doi:10.1186/s12893-026-04008-6)
Supplement: Supplementary file 1 [file 12893_2026_4008_MOESM1_ESM.zip › Supplementary Table 3.docx]

**Supplementary Table 3 Ridge-penalized logistic regression output**

| Model | Term | Coefficient | OR per SD | 95% CI | Approx. P-value |
| --- | --- | --- | --- | --- | --- |
| Primary preoperative/peri-induction model | Intercept | -1.187 | 0.305 | 0.185-0.504 | <0.001 |
|  | pH | -0.615 | 0.541 | 0.355-0.822 | 0.004 |
|  | Lactate | 0.207 | 1.230 | 0.838-1.805 | 0.291 |
|  | Heart rate at beginning of surgery | 0.201 | 1.222 | 0.828-1.804 | 0.313 |
|  | Pulmonary valve VMAX | 0.370 | 1.448 | 0.973-2.153 | 0.068 |
|  | PASP at beginning of surgery | 0.210 | 1.233 | 0.840-1.811 | 0.285 |
| Secondary intraoperative monitoring model | Intercept | -1.445 | 0.236 | 0.127-0.439 | <0.001 |
|  | pH | -1.304 | 0.272 | 0.131-0.564 | <0.001 |
|  | Lactate | 0.262 | 1.300 | 0.759-2.227 | 0.340 |
|  | Heart rate at beginning of surgery | 0.299 | 1.349 | 0.779-2.336 | 0.286 |
|  | Pulmonary valve VMAX | 0.502 | 1.652 | 0.882-3.094 | 0.117 |
|  | Highest intraoperative PASP | 1.005 | 2.731 | 1.421-5.248 | 0.003 |

*All continuous predictors were standardized before model fitting. ORs are reported per one standard deviation increase in the standardized predictor. The p-values are approximate Wald p-values from the penalized model output and should be interpreted cautiously in this small exploratory cohort.*
