# Supplementary material for: Perioperative factors associated with intraoperative circulatory decompensation during lung transplantation under initial VV-ECMO support: an exploratory single-center study
Source: BMC Surg. 2026 Jul 4;26:649. doi: 10.1186/s12893-026-04008-6 (PMC13617856; doi:10.1186/s12893-026-04008-6)
Supplement: Supplementary file 1 [file 12893_2026_4008_MOESM1_ESM.zip › Supplementary Table_1.docx]

**Supplementary Table 1. Preoperative characteristics and intraoperative conversion reasons of the study cohort**

| **Patient No** | **Primary Disease** | **First Operative Side** | **Conversion Timing** | **Reason for Conversion** | **LAS Score** | **Oxygen Concentration (FiO₂)** | **6-Minute Walk Distance** |
| --- | --- | --- | --- | --- | --- | --- | --- |
| 1 | COPD | Left | During second lung PA clamping | Graft pulmonary edema; pulmonary artery pressure increased to 80 mmHg; systemic circulation could not be maintained | 70.41 | 0.40 | Not performed |
| 2 | COPD | Left | During second lung PA clamping | Pulmonary artery pressure increased to 75 mmHg; systemic circulation instability | 64.70 | 0.35 | Not performed |
| 3 | Bronchiectasis | Left | During first lung PA clamping | RV failure; pulmonary artery pressure increased to 50 mmHg; systemic circulation instability | 29.99 | 0.45 | Not performed |
| 4 | Bronchiolitis obliterans | Right | During second lung PA clamping | RV failure; pulmonary artery pressure increased to 50 mmHg; systemic circulation instability | 30.05 | 0.40 | Not performed |
| 5 | COPD | Left | During first lung PA clamping | Pulmonary artery pressure increased to 80 mmHg; systemic circulation instability | 83.18 | 0.45 | Not performed |
| 6 | Interstitial lung disease | Right | During second lung PA clamping | Pulmonary artery pressure elevation with hemodynamic instability | 38.70 | 0.33 | Not performed |
| 7 | Interstitial lung disease | Right | During second lung PA clamping | Pulmonary hypertension with circulatory instability | 35.82 | 0.40 | Not performed |
| 8 | Interstitial lung disease | Left | During second lung PA clamping | Pulmonary hypertension and circulatory instability | 37.86 | 0.28 | 215 m |
| 9 | Interstitial lung disease | Left | During second lung PA clamping | Severe pulmonary hypertension during PA clamping | 64.48 | 0.50 | Not performed |
| 10 | Bronchiectasis | Right | During second lung PA clamping | Pulmonary hypertension with systemic instability | 41.70 | 0.33 | Not performed |
| 11 | Pneumoconiosis | Right | During second lung PA clamping | Pulmonary hypertension with circulatory instability | 29.68 | 0.55 | Not performed |
| 12 | Interstitial lung disease | Left | During second lung PA clamping | Hemodynamic instability during PA clamping | 84.81 | 0.30 | Not performed |
| 13 | Interstitial lung disease | Right | During second lung PA clamping | Pulmonary hypertension with RV dysfunction | 33.74 | 0.35 | 434 m |
| 14 | Interstitial lung disease | Left | During second lung PA clamping | Pulmonary hypertension with circulatory instability | 29.37 | 0.29 | Not performed |
| 15 | Interstitial lung disease | Left | During second lung PA clamping | Pulmonary hypertension during PA clamping | 29.40 | 0.41 | Not performed |
| 16 | Interstitial lung disease | Left | During second lung PA clamping | Severe pulmonary hypertension | 91.79 | 0.60 | Not performed |
| 17 | Interstitial lung disease | Right | During second lung PA clamping | Hemodynamic instability with pulmonary hypertension | 38.76 | 0.29 | 273 m |
| 18 | Interstitial lung disease | Right | During second lung PA clamping | Pulmonary hypertension with systemic instability | 42.06 | 0.37 | Not performed |
| 19 | Interstitial lung disease | Left | During second lung PA clamping | Pulmonary hypertension during PA clamping | 33.84 | 0.50 | Not performed |
| 20 | Interstitial lung disease | Left | During second lung PA clamping | Severe pulmonary hypertension | 27.70 | 0.60 | Not performed |
| 21 | Interstitial lung disease | Left | During second lung PA clamping | Pulmonary hypertension with hemodynamic compromise | 54.00 | 0.32 | Not performed |
| 22 | Interstitial lung disease | Left | During second lung PA clamping | Severe pulmonary hypertension | 84.62 | 1.00 | Not performed |
| 23 | Interstitial lung disease | Left | During second lung PA clamping | Severe pulmonary hypertension | 34.30 | 1.00 | Not performed |
| 24 | Interstitial lung disease | Right | During second lung PA clamping | Pulmonary hypertension with circulatory instability | 29.04 | 0.39 | Not performed |
| 25 | Interstitial lung disease | Left | During second lung PA clamping | Pulmonary hypertension with circulatory instability | 28.35 | 0.40 | Not performed |

Abbreviations: LAS, Lung Allocation Score; FiO₂, fraction of inspired oxygen; PA, pulmonary artery.
