# Supplementary material for: Perioperative factors associated with intraoperative circulatory decompensation during lung transplantation under initial VV-ECMO support: an exploratory single-center study
Source: BMC Surg. 2026 Jul 4;26:649. doi: 10.1186/s12893-026-04008-6 (PMC13617856; doi:10.1186/s12893-026-04008-6)

Figure 1. Intraoperative VV-to-VA Conversion Algorithm

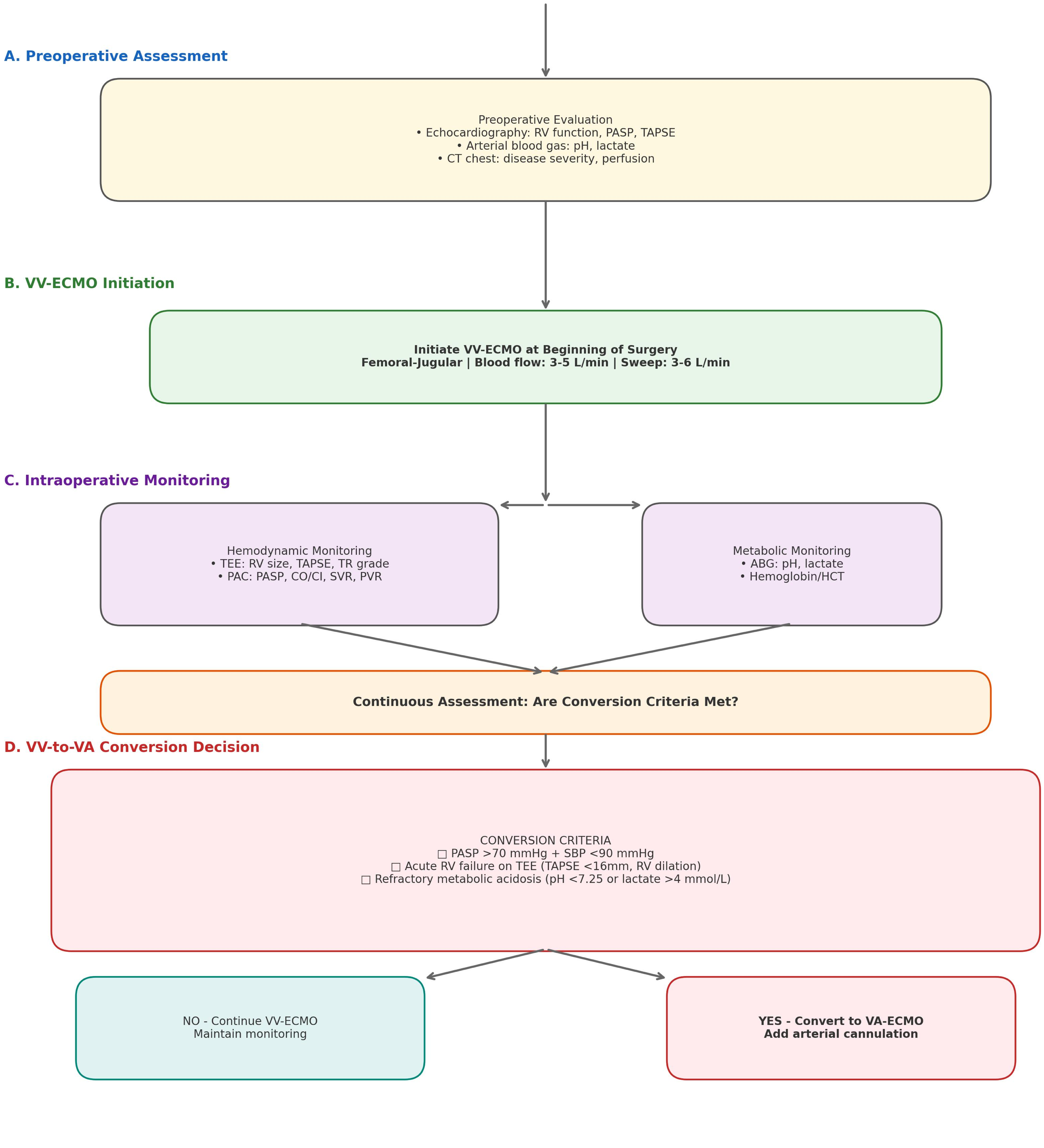

Supplement: Supplementary file 1 [file 12893_2026_4008_MOESM1_ESM.zip › supplementary figure 1.pdf]
