# Supplementary material for: Perioperative factors associated with intraoperative circulatory decompensation during lung transplantation under initial VV-ECMO support: an exploratory single-center study
Source: BMC Surg. 2026 Jul 4;26:649. doi: 10.1186/s12893-026-04008-6 (PMC13617856; doi:10.1186/s12893-026-04008-6)
Supplement: Supplementary file 1 [file 12893_2026_4008_MOESM1_ESM.zip › supplementary figure 2.pdf]

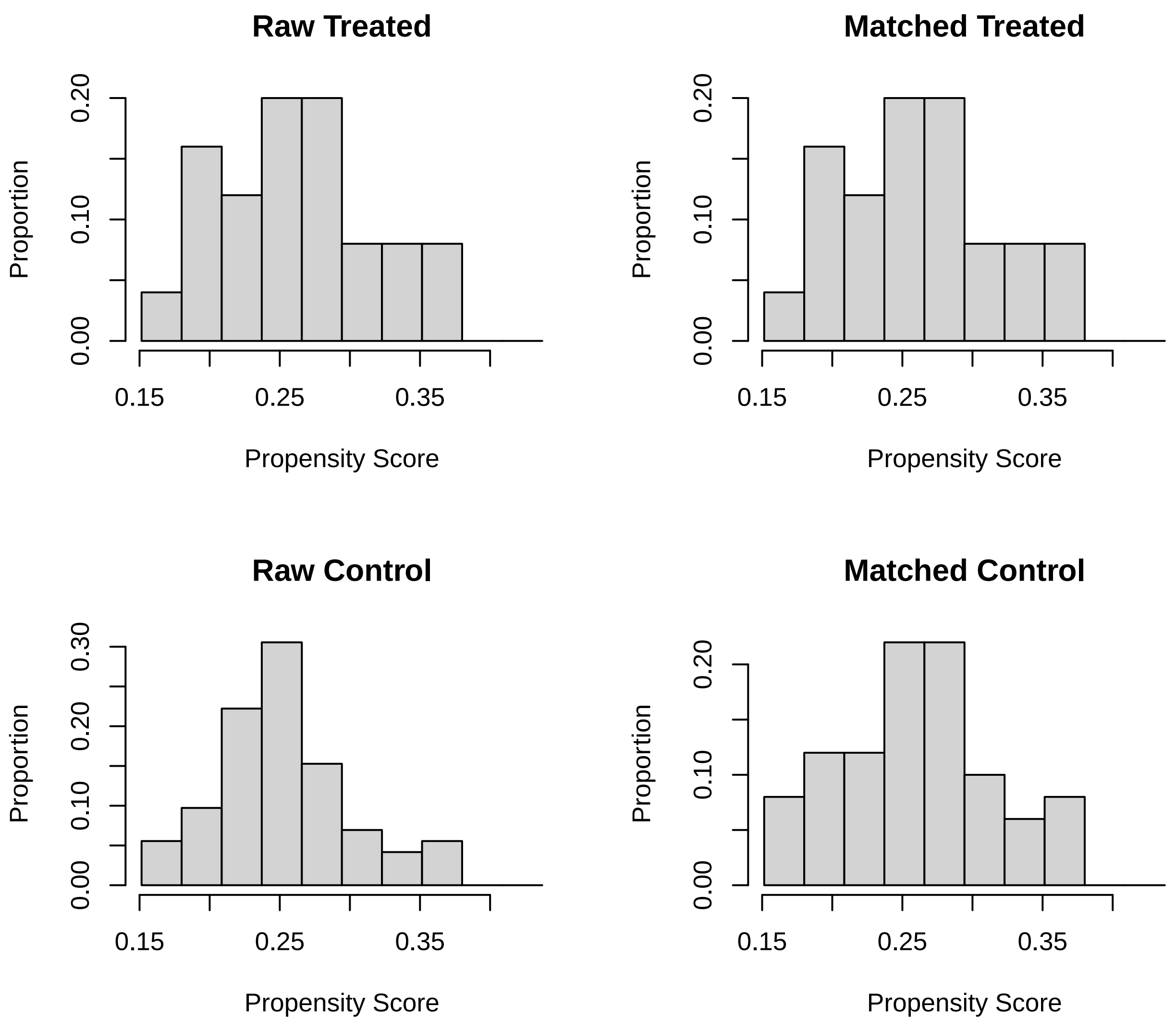
Raw Treated
Matched Treated
Raw Control
Matched Control
Proportion
Propensity Score
0.00
0.10
0.20
0.30
0.15
0.25
0.35
