# Supplementary material for: Perioperative factors associated with intraoperative circulatory decompensation during lung transplantation under initial VV-ECMO support: an exploratory single-center study
Source: BMC Surg. 2026 Jul 4;26:649. doi: 10.1186/s12893-026-04008-6 (PMC13617856; doi:10.1186/s12893-026-04008-6)
Supplement: Supplementary file 1 [file 12893_2026_4008_MOESM1_ESM.zip › supplementary figure 3.pdf]

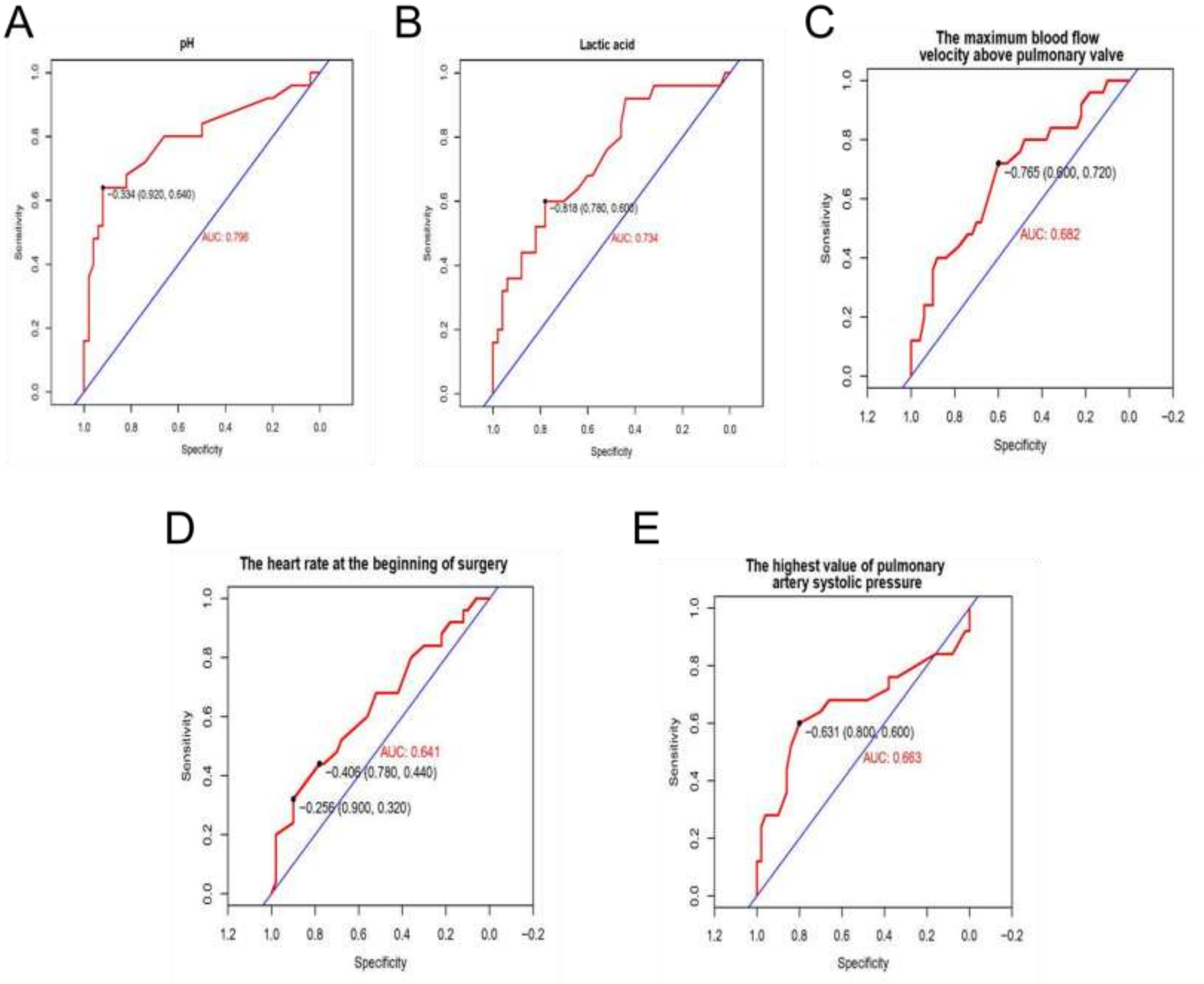

A
pH
−0.334 (0.920, 0.640)
AUC: 0.796
Sensitivity
Specificity
B
Lactic acid
−0.818 (0.780, 0.600)
AUC: 0.734
Sensitivity
Specificity
C
The maximum blood flow velocity above pulmonary valve
−0.765 (0.600, 0.720)
AUC: 0.682
Sensitivity
Specificity
D
The heart rate at the beginning of surgery
AUC: 0.641
−0.406 (0.780, 0.440)
−0.256 (0.900, 0.320)
Sensitivity
Specificity
E
The highest value of pulmonary artery systolic pressure
−0.631 (0.800, 0.600)
AUC: 0.663
Sensitivity
Specificity
